# Optogenetic conditioning of paradigm and pattern discrimination in the rat somatosensory system

**DOI:** 10.1371/journal.pone.0189439

**Published:** 2017-12-21

**Authors:** Kenta Abe, Hiromu Yawo

**Affiliations:** 1 Department of Development Biology and Neuroscience, Tohoku University Graduate School of Life Science, Sendai, Japan; 2 Center for Neuroscience, Tohoku University Graduate School of Medicine, Sendai, Japan; Hokkaido Daigaku, JAPAN

## Abstract

The rodent whisker-barrel cortical system is a model for studying somatosensory discrimination at high spatiotemporal precision. Here, we applied optogenetics to produce somatosensory inputs in the whisker area using one of transgenic rat lines, W-TChR2V4, which expresses channelrhodopsin-2 (ChR2) in the mechanoreceptive nerve endings around whisker follicles. An awake W-TChR2V4 rat was head-fixed and irradiated by blue LED light on the whisker area with a paradigm conditioned with a reward. The Go task was designed so the rat is allowed to receive a reward, when it licked the nozzle within 5 s after photostimulation. The No-go task was designed so as the rat has to withhold licking for at least 5 s to obtain a reward after photostimulation. The Go-task conditioning was established within 1 hr of training with a reduction in the reaction time and increase of the success rate. To investigate the relationship between the spatiotemporal pattern of sensory inputs and the behavioral output, we designed a multi-optical fiber system that irradiates the whisker area at 9 spots in a 3×3 matrix. Although the Go-task conditioning was established using synchronous irradiation of 9 spots, the success rate was decreased with an increase of the reaction time for the asynchronous irradiation. After conditioning to the Go task, the rat responded to the blue LED flash irradiated on the barrel cortex, where many neurons also express ChR2, or photostimulation of the contralateral whisker area with a similar reaction time and success rate. Synchronous activation of the peripheral mechanoreceptive nerves is suggested to drive a neural circuit in the somatosensory cortex that efficiently couples with the decision. Our optogenetic system would enable the precise evaluation of the psychophysical values, such as the reaction time and success rate, to gain some insight into the brain mechanisms underlying conditioned behaviors.

## Introduction

The behavioral responses of an animal are determined by the brain through the integration of various sensory inputs from the body [1]. For example, when the skin contacts with an object, the information is sensed by the peripheral mechanoreceptive organs such as Meissner corpuscles and Merkel corpuscles to generate action potentials in the mechanoreceptive nerve endings of neurons in the dorsal root ganglion (DRG) or the trigeminal ganglion (TG). The action potentials are conducted to the contralateral somatosensory cortex via the medulla and thalamus to generate the sense of touch. As this pathway is topographically organized, the animal recognizes where in the body the sensation originated.

Rodents use their whiskers/vibrissae to collect information about objects: position, size, shape and texture [2–4]. The principal whiskers are two-dimensionally arrayed on the snout and richly innervated by the maxillary branch of the TG nerve at their follicles. The deflection of each whisker evokes action potentials in the nerve endings which are conducted to the relatively large field of the primary somatosensory cortex (S1) called the “barrel” through the brainstem and thalamus. The thalamo-cortical projections are thus somatotopically organized in layer 4 so as to map the whisker arrangement of the contralateral side, although some of them have broad receptive fields. Therefore, the whisker-barrel cortical system has been an ideal model for studying somatosensory discrimination at high spatiotemporal precision [5].

To reveal the neural basis underlying object discrimination, we adopted a psychophysical analysis using optogenetics in combination with behavioral tests to produce various sensory inputs in the whisker area on the snout of a rat because of its high spatiotemporal precision [6]. Previously, we generated several transgenic lines of rats that express channelrhodopsin-2 (ChR2), an algal light-driven cation channel [7–8], under the control of thy1.2 promoter [9]. In one of them, named W-TChR2V4, ChR2 was expressed in the large mechanoreceptive neurons in the DRG and TG, but not in the nociceptive neurons [10]. It was also expressed in the mechanoreceptive nerve endings innervating richly around the follicles of whiskers. Therefore, the blue light irradiated on the whisker area is well expected to depolarize the mechanoreceptive nerve endings selectively to evoke action potentials through the epidermis. Indeed, the electrical and fMRI responses were generated in the barrel field of the contralateral somatosensory cortex in a manner dependent on the whisker area irradiation by blue LED light [11].

In the present study, the optogenetic inputs were given in the whisker area of the W-TChR2V4 rat with spatiotemporal precision. Each input was quantitatively related to the behavioral response of the rat with a paradigm conditioned with a reward [12–15]. We found that the rat discriminated among changes in the spatiotemporal pattern of inputs. Synchronous activation of a certain number of peripheral mechanoreceptive nerves is suggested to drive a kind of neural circuit in the somatosensory cortex that efficiently couples with the decision.

## Materials and methods

### Animal

All animal experiments were carried out in accordance with the animal experiment protocol approved by Tohoku University Committee for Animal Experiments (Approval No. 2017LsA-001) and were carried out in accordance with the guidelines for Animal Experiments and Related Activities of Tohoku University as well as the guiding principles of the Physiological Society of Japan and the National Institutes of Health (NIH), USA. The number of animals in this study was kept to a minimum and, when possible, all animals were anesthetized to minimize their suffering. Animals had access to food and water *ad libitum* and were kept under a 12 hour light-dark cycle.

The transgenic rats expressing ChR2-Venus under the control of Thy1.2 promoter in the nervous system (W-TChR2V4, ChR2 rats, NBRP#0685, National BioResource Project—Rat, Kyoto University, Kyoto, Japan) were originally produced from the zygotes of Wistar rats and have been bred for about ten years under mating with Wistar rats [9–11]. Adult ChR2 rats (180g-280g) and wild-type Wistar rats (Wt: 180–230 g) were used for the experiments. Briefly they were anesthetized with a mixture (1 ml/kgBW) of ketamine (50 mg/ml, Daiichi Sankyo Co. Ltd., Tokyo, Japan) and xylazine hydrochloride (10 mg/ml, Sigma-Aldrich, St. Louis, MO, USA). A head plate (CFR-1, Narishige, Japan) was surgically attached to the skull of each rat with tiny anchor screws (M1.4×3) and dental resin cement (Super Bond C&B, Sun Medical, Moriyama City, Japan), while its body temperature was maintained at 37°C using a homeothermic heating pad. Three-to-four days after recovery from the surgery, the rats were deprived of drinking water over 48 hrs in their home cage, where food was available *at libitum*, and were adapted to a stainless steel cylinder into which they introduced their body during the following behavioral experiments [16–18].

Some animals were visually deprived at both sides [18]. Briefly, under ketamine-xylazine anesthesia, a small incision was made with surgical scissors in the conjunctiva beginning inferior to the globe and around the eye temporally. In order to protect the underlying extraocular muscles from injury, precautions were taken not to cut the tissue too deeply. When the posterior face of the globe was exposed, the optic nerve was amputated under visualization with minimal damage to the vasculatures.

### Optical system

All whiskers as well as the intervibrissal fur of the right side were trimmed off from the rat snout. The whisker area was irradiated by a light pulse (50 ms, 10 ± 2 mW/mm^2^ at the surface of skin) from either the blue LED (470 nm, LXML-PB01-0040, Philips Lumileds Lighting Co. San Jose, CA, USA) or red LED (625 nm, LXML-PD01-0040, Philips Lumileds Lighting Co.). The leakage of LED light was minimized by setting it close to the snout (~5 mm).

The spatiotemporally patterned light cue was made by a multi-optical fiber system consisting of 9 plastic optical fibers (750 μm, GCK-30E, Mitsubishi Rayon, Co. Ltd., Tokyo, Japan) arrayed in a 3×3 matrix (center-to-center distance, 3 mm) on a piece of acrylic board (1×1 cm) that was set ~5 mm above the whisker area. The other end of each optical fiber was connected to the LED light source (470 nm, FCS-0470-000, Mightex Systems, Toronto, Canada), the on-off function of which was controlled by three LED drivers (SLC-SA04-US, Mightex Systems) and a computer. The power of irradiation from each fiber end was ~10 mW/mm^2^ at the surface of whisker area. Each light spot made by a fiber covered at least 1 whisker follicle.

To irradiate the barrel cortex directly, a small circular region (diameter, 2–3 mm) on the skull (5 mm lateral, 3 mm rostral from the bregma) was scraped off until it became so thin that the blood vessels running underneath could be observed faintly. The surface of the brain was clearly visualized by setting a small tube on the hole with dental cement and dropping light mineral oil (Sigma-Aldrich, St. Louis, MO, USA) in it to raise the refractive index (1.467). The barrel cortex was irradiated by the light pulse (50 ms, 2–3 mW/mm^2^ at the surface of the brain) from blue LED (470 nm, LXML-PB01-0040, Philips Lumileds Lighting Co.) placed over it at 4–5 mm.

### Behavioral test

**Fig 1:**
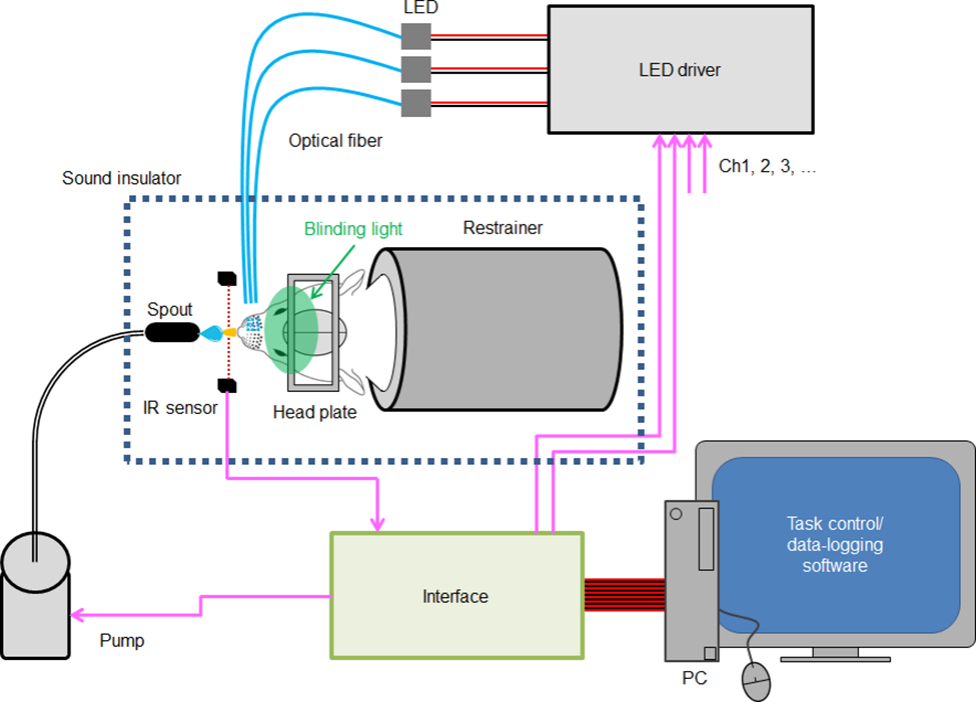
Behavioral test system. The head of the fully awake rat was fixed with a head plate in a stereotaxic frame while its body was in the restrainer. The lick was detected by the IR sensor in front of its mouth. When the lick was judged to be correct, a drop of sweet water was pumped out from the spout as a reward. The photostimulation of the whisker area was made by a pulse of LED light the on-off function of which was controlled by three LED drivers. The spatiotemporally patterned light cue was made by a 3×3 array of 9 plastic optical fibers, the other end of which was connected to the LED light source. These devices were under control of the software through an interface while logging the sensor signal. The rat was isolated from environmental sound and blinded by green LED light on both eyes to prevent the pump sound or leaked light from being utilized as the learning cue.

The behavioral test system (Fig 1) consisted of a stereotaxic frame (Model 900, David Kopf Instruments, Tujunga, CA, USA) in which the head of the rat, while awake, was fixed with a head plate, a liquid feed pump, three LED drivers (SLC-SA04-US, Mightex Systems), a computer, an interface controller device and task control software (TaskForcer, O’HARA Co, Ltd., Tokyo, Japan) [16, 17]. A spout was set in front of the rat’s mouth and water containing 0.1% saccharin (5 μl) was pumped out as a reward. When the rat licked the nozzle, it was detected by the infrared radiation sensor. Throughout the experiment, the rat was isolated from environmental sound and blinded by green LED light on both eyes to prevent the pump sound or leaked light from being utilized as the learning cue. Each training session was a 0.5-*hr* series of tasks sequentially applied with an inter-task break (ITB) of 10 ± 3 s. The ±3-s variability of ITB was given randomly so that the rat could not expect the timing of rewards.

### Date analysis

All data were logged by the manufacturer’s software (Operant Task Studio, O’HARA Co, Ltd.) and off-line analyzed by the home-made programs. All data in the text and figures were expressed using box-and-whisker plots, in which the box indicated the upper/lower quartile, the line the maximum/minimum and the mark the median of the data. The statistical evaluation was conducted using Wilcoxon signed rank test for the statistical significance for paired data, using Mann-Whitney *U*-test for unpaired data and using Kruskal-Wallice test by ranks for data in order unless otherwise noted. The numbers of animals used for the statistics (n) are shown in the figure legends. It was judged as statistically insignificant when P > 0.05.

## Results

### Go task conditioning with light cue

**Fig 2:**
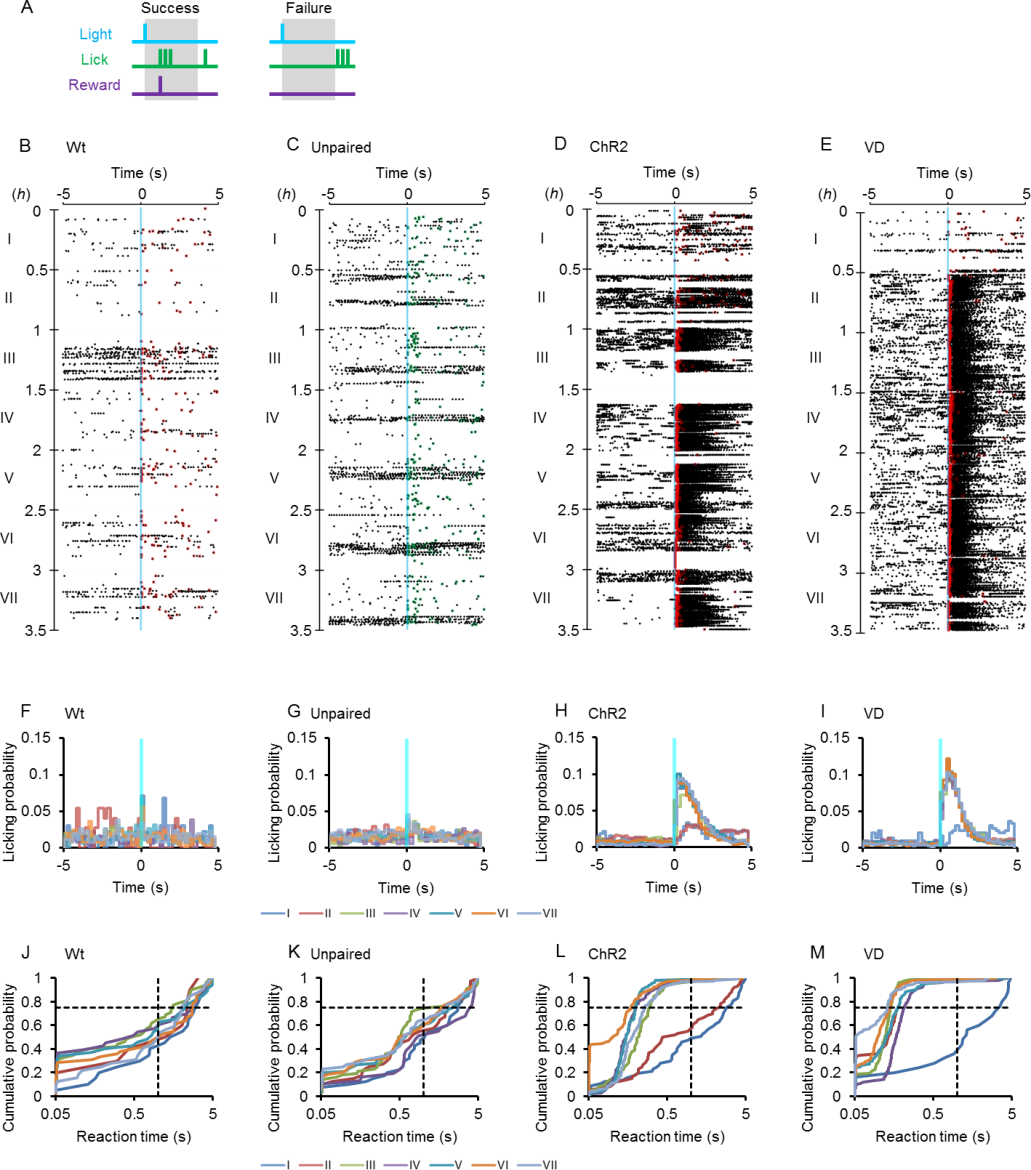
Optogenetic conditioning of the Go task. **A,** Go task training paradigm. The rat was rewarded when it licked the nozzle within the 5-s judgement time window (success, left), but unrewarded if not (failure, right). **B-E,** Sample raster plots of licking events, which were aligned to the onset of blue light cue (blue vertical line) during the Go task training sessions (I~VII) of the control wild-type rat (**B**, Wt), the control W-TChR2V4 (ChR2) rat unpaired with reward (**C**, Unpaired), the ChR2 rat paired with reward (**D**, ChR2) and the visually deprived ChR2 rat (**D**, VD), respectively. The overlaid squares indicate the first lick with (red) or without rewards (green). **F-I,** Histograms of licking probability before 5 s and after 5s of the onset of blue light cue (blue vertical line) for the data shown in B-E: the control wild-type rat (**F**), the unpaired control ChR2 rat (**G**), the paired ChR2 rat (**H**) and the visually deprived ChR2 rat (**G**). **J-M,** Cumulative probability plots of the reaction time for the data shown in B-E. Note that the horizontal axis is scaled logarithmically to show the traits of early events. The broken lines indicate the cumulative probability at 0.75 (the third quartile) and the reaction time at 1 s. Although distributional differences were not significant among training sessions for the control rats (**J** and **K**), the difference between early sessions (I) and later ones (III~VII) was highly significant (P < 10^−9^, Kolmogorov-Smirnov test) for the ChR2 rats with and without VD (**L** and **M**).

To test the ability of a rat to learn the optogenetic input on the whisker area as a conditional stimulation to evoke a behavior, a Go task training paradigm was designed to award the sweet water once when the rat lick the nozzle within 5 s after the onset of a brief (50 ms) blue light cue (Fig 2A, success). However, the rat was not rewarded when it did not lick the nozzle within this judgement time window (failure). During 7 successive training sessions (I~VII), each of which consisted of the Go task training for 0.5 h with an inter-task break (ITB) of 10 ± 3 s, wild-type (Wt) rats occasionally received the reward after the blue light clue, but showed no tendency to relate it to the cue (Fig 2B). As shown in Fig 2F, the licking probability was distributed evenly during the task period throughout the progress of the sessions. In contrast, the W-TChR2V4 (ChR2) rats were soon conditioned, although they did not succeed frequently when they were naïve to the training (Fig 2D). Indeed, an increase of the licking probability was observed immediately after the blue light cue (Fig 2H). When the reaction time was measured between the onset of light cue and the first licking event, its distribution shifted to the smaller values with the progress of training sessions and the majority of events occurred within 0.5 s (Fig 2L). On the other hand, such a shift in the distribution was not observed in control experiments using Wt rats (Fig 2J) or in an unpaired control group of ChR2 rats for which the blue light cue was not conditioned with a reward (Up, Fig 2C, 2G and 2K).

**Fig 3:**
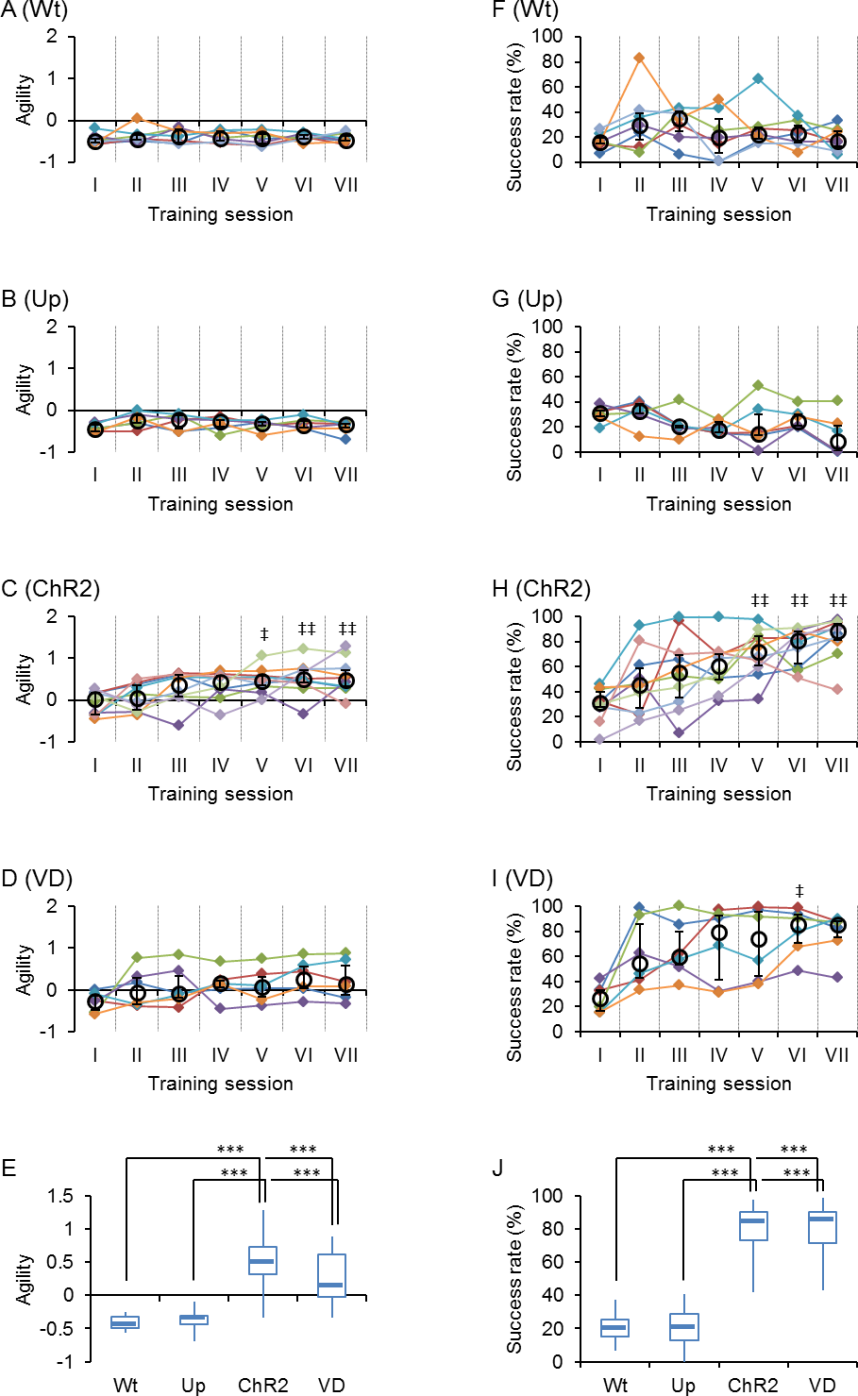
Quantitative analysis of behavioral responses. **A-D,** Summary of changes of agility during Go task training; the control wild-type rats (**A**, Wt, n = 7), the control unpaired ChR2 rats (**B**, Up, n = 6), the paired ChR2 rats (**C**, ChR2, n = 10) and the visually deprived ChR2 rats (**D**, VD, n = 6). The black circle and bar indicate median and higher/lower quartile, while each colored mark indicates an individual rat. **E**, Box-and-whisker plots comparing the agility among Wt (n = 7), Up (n = 6), ChR2 (n = 10) and VD (n = 6) during sessions VI and VII. **F-I,** Similar to A-D, but the changes in the success rate during the Go task training are summarized. **J**, Box-and-whisker plots comparing the success rate among Wt (n = 7), Up (n = 6), ChR2 (n = 10) and VD (n = 6) during sessions VI and VII. ***, P < 0.0005, Mann-Whitney *U*-test. ^‡^, P < 0.05, ^‡‡^, P < 0.01, Kruskal-Wallice test by ranks against session I.

These differences were sensitively compared by the time when the cumulative probability reached to 0.75 (the third quartile of reaction time distribution, *RT*_75_) because it was usually larger than 1 s in the control groups (Wt and Up), but became less than 1 s with the establishment of conditioning. In the following analyses we used the logarithmic reciprocal of this value, -log(*RT*_75_), which we named “agility” to evaluate the reaction speed of an animal quantitatively (S1 Fig). The agility is negative when 75% of the cue trials were accompanied by the first lick after 1 s and positive if within 1 s. Indeed, the agility was almost negative throughout sessions of any rats in the control groups (99%, Wt and Up, Fig 3A and 3B). On the other hand, it significantly increased with the progress of the training sessions for the ChR2 rats (Fig 3C and 3E). The success rate, the chance of licking within the judgement time window with/without reward, was also measured for each session (S1 Fig) and compared between the ChR2 rat group and the control groups (Fig 3F–3H). It significantly increased with the progress of the training sessions for the ChR2 rats, whereas it was usually less than 50% for the control groups (Fig 3J).

Some of the ChR2 rats were visually deprived (VD) by severing their optic nerves on both sides. During 7 successive Go task training sessions, each of the VD rats was soon conditioned with an increase of the licking probability immediately after the blue cue light (Fig 2E and 2I). Similar to the ChR2 rats without VD, the reaction time showed a tendency to shift to smaller values with the progress of the training sessions (Fig 2M). Indeed, the agility of the VD rats was significantly greater than that of the control groups (Wt and Up) at later training sessions (Fig 3D and 3E). Similarly, the success rate of VD rats increased significantly in the later training sessions (Fig 3I and 3J).

### Tests for light dependency

**Fig 4:**
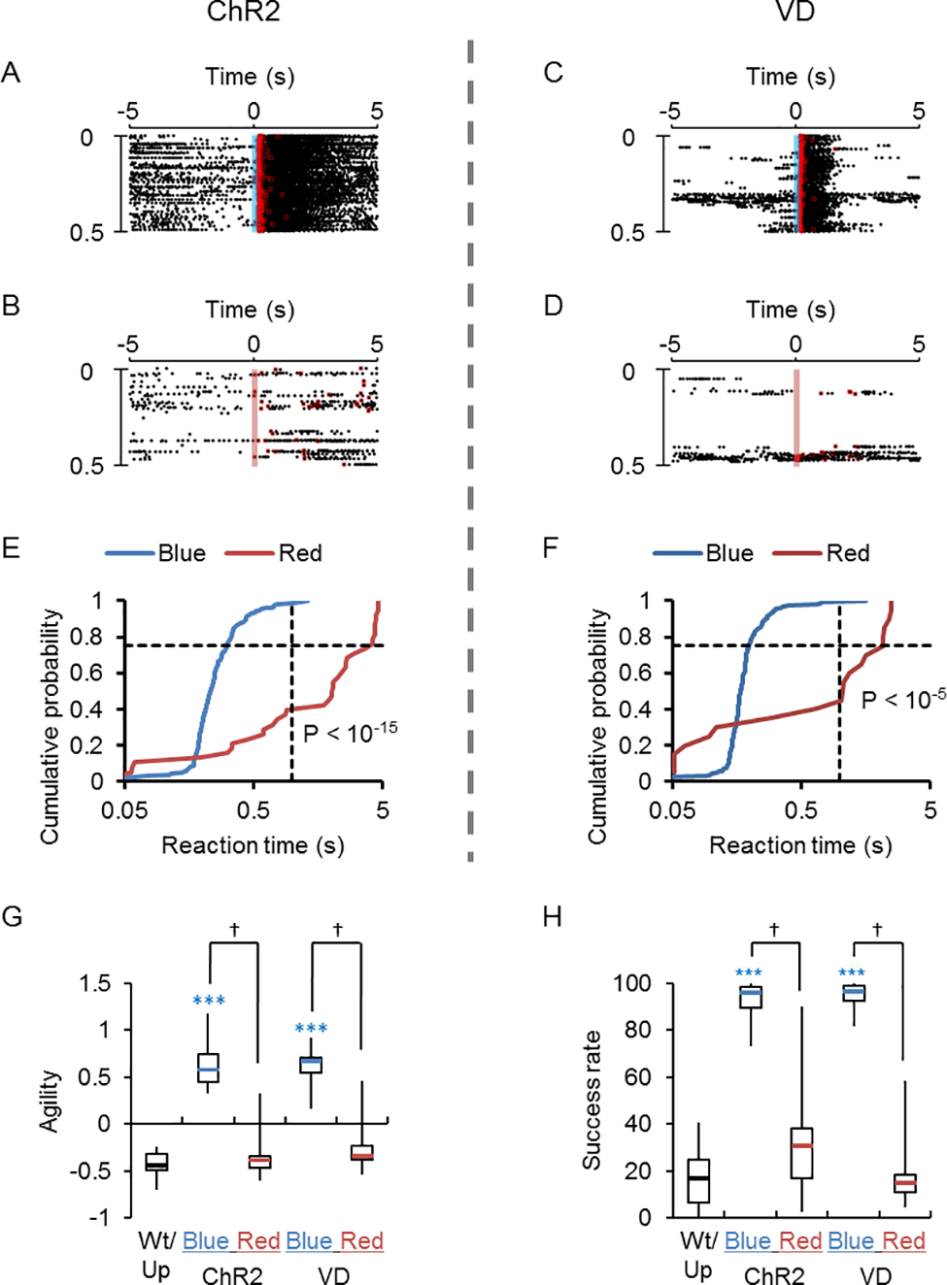
Color dependency tests of the light cue. **A-B,** Sample raster plots of licking events, which were aligned to the blue light cue (**A**, blue vertical line) or red light cue (**B**, red vertical line) after Go task conditioning had been established for a W-TChR2V4 rat (ChR2). The red squares indicate the rewards. **C-D,** Similar to A and B, but for another ChR2 rat that was visually deprived (VD). **E-F,** Cumulative probability plots of the reaction time for the data shown in A-B (**E**) and C-D (**F**). The difference between experiments with blue and red light cues was highly significant (P < 10^−5^, Kolmogorov-Smirnov test) for the ChR2 (**E**) and the VD (**F**). **G,** Comparison of agility to the color of light cues. Box-and-whisker plots from left to right; control groups (Wt/Up, black mark, n = 13), blue for the ChR2 (blue mark, n = 7), red for the ChR2 (red mark, n = 7), blue for the VD (blue mark, n = 6) and red for the VD (red mark, n = 6). **H,** Similar to G, but box-and-whisker plot comparison of success rate according to the color of the light cues. ***, P < 0.0005, Mann-Whitney *U*-test for the control. ^†^, P < 0.05, Wilcoxon signed rank test between blue and red light cues.

The possibility that the ChR2 rat sensed the blue light at their whisker area as a cue of conditioning was tested by two additional experiments: (1) the use of red LED light as a cue signal and (2) the shading of the whisker area from the blue light cue. Although the first licking was synchronized with the blue light cue after establishing the conditioning (Fig 4A and 4E), it did not occur when the color of cue signal light was switched to red (Fig 4B and 4E). Indeed, the agility was significantly reduced to the level of control groups (Wt and Up, Fig 4G). The switching of cue signal light from blue to red also significantly reduced the success rate (Fig 4H). Similar effects of the signal color switching were observed with the VD rats with significant changes in the agility and success rate (Fig 4C, 4D, 4F, 4G and 4H).

**Fig 5:**
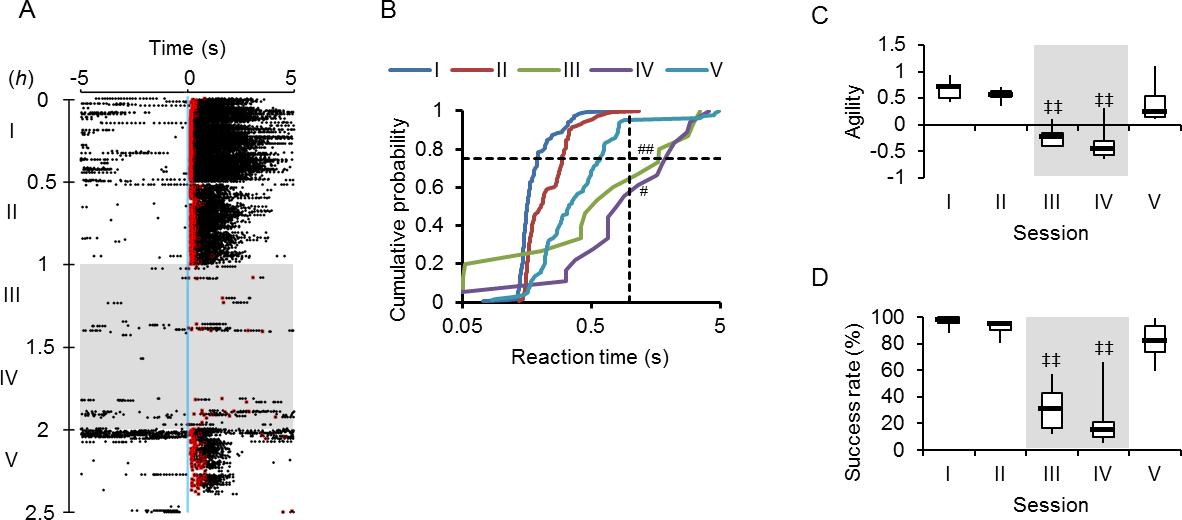
Regional shading tests. **A,** Sample raster plots of cue-aligned licking events for a series of training sessions (I~V) after the establishment of Go task conditioning; the red squares indicate the rewards. The whisker area was regionally shaded at sessions III and IV (gray background). **B,** Cumulative probability plots of the reaction time for the data shown in A. ^##^, P < 10^−4^ (Kolmogorov-Smirnov test) between session III and before (I or II). ^#^, P < 10^−3^ (Kolmogorov-Smirnov test) between session IV and after (V). **C,** Summary of agility through consecutive sessions (n = 7); the shaded sessions III and IV were backgrounded with gray. D, Summary of success rate (n = 7). ^‡^, P < 0.05, ^‡‡^, P < 0.01, Kruskal-Wallice test by ranks to sessions I~II (session III) or to session V (session IV).

In another series of experiments, the whisker area was regionally shaded from the blue cue light by the placement of a small aluminum patch. As shown in Fig 5A, once established, conditioning to the blue light cue (sessions I and II) was transiently suppressed upon light shade (III and IV), but was recovered immediately without the shade (V). The change of the behavioral response was quantitatively expressed as the distributional shift of the reaction time (Fig 4B) and evaluated by the agility, which was significantly reduced by shading (Fig 4C). Similarly, the success rate was reversibly reduced by shading (Fig 4D).

### Retention and extinction of conditioned response

**Fig 6:**
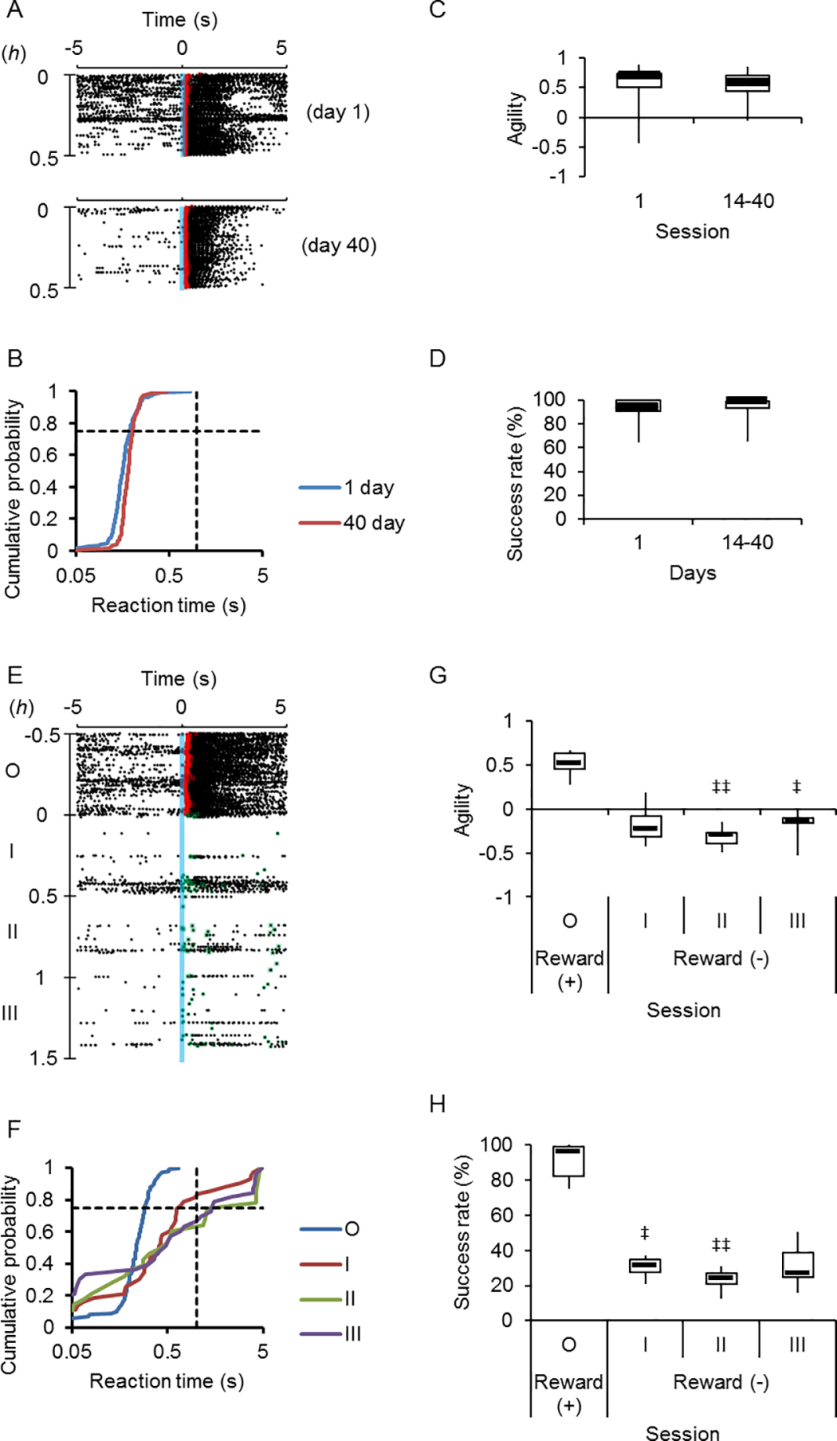
Retention and extinction of conditioned response. **A,** Sample raster plots of the retention test. The Go task conditioning was established at the last session of the first training day (top). A similar conditioned response was evoked even during the first session of the Go task after the non-training period of 40 days (bottom). The red squares indicate the first licks after light cues with rewards. **B,** Cumulative probability plots of the reaction time for the data shown in A. **C,** Summary of the agility (n = 5 at 14 days and n = 6 at 40 days). **D**, Summary of the success rate (n = 5 at 14 days and n = 6 at 40 days). **E,** Sample raster plots of the extinction test. The Go task conditioning (session O) was followed by sessions without rewards (I~III). The red squares indicate the first licks after light cues with rewards, whereas the green squares indicate the first licks without rewards. **F,** Cumulative probability plots of the reaction time for the data shown in E. **G,** Summary of the agility through consecutive sessions (n = 6). **H**, Summary of the success rate (n = 6). ^‡^, P < 0.05, ^‡‡^, P < 0.01, Kruskal-Wallice test by ranks.

The blue light-conditioned licking response of the ChR2 rats was retained even after an interval of days with no training (Fig 6A). Although variable among experiments, the reaction time during the first session of the Go task after the non-training period of 14–40 days was similar to that during the last session at the first training day with insignificant difference in the agility and the success rate (Fig 6B–6D). On the other hand, the blue light no longer induced the licking behavior when it was not associated with the reward (Fig 6E). The change in the behavioral response was again quantitatively expressed as a distributional shift of the reaction time between the blue light cue and the first lick (Fig 6F) and evaluated by the agility, which was significantly reduced during the sessions (II and III) with no reward (Fig 6G). Similarly, the success rate was reduced to less than 50% even during the earliest session (I) with no reward (Fig 6H).

### Switching from Go to Nogo task

**Fig 7:**
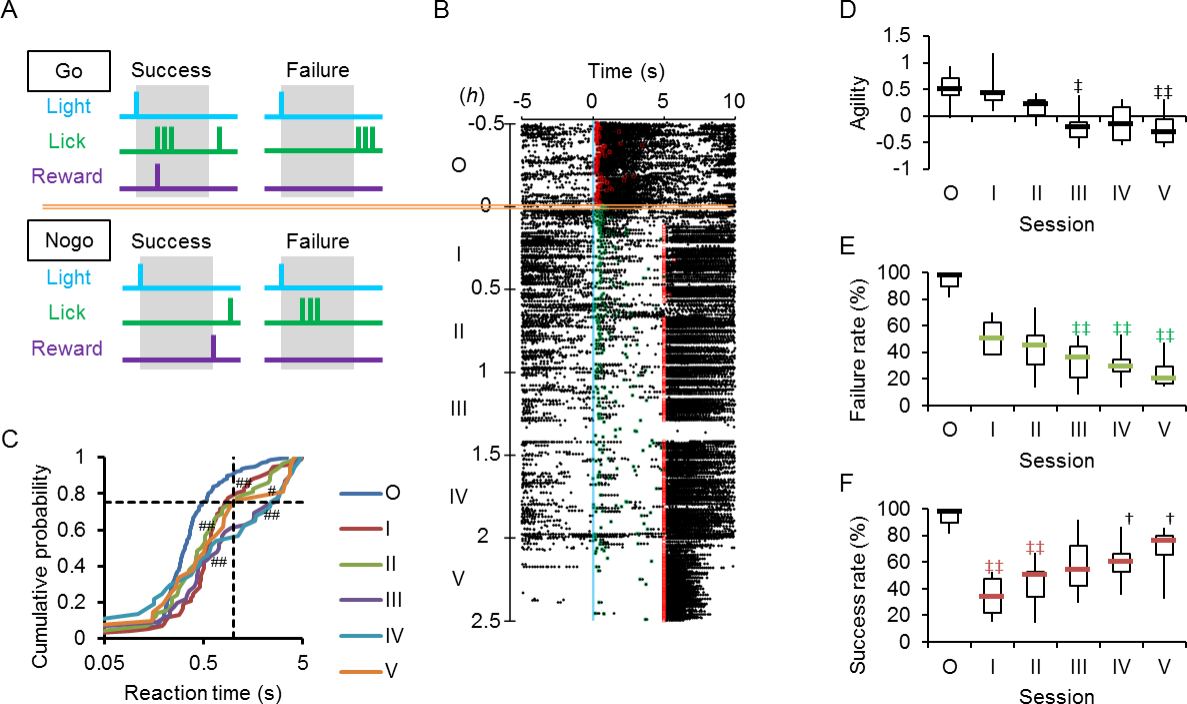
Go to Nogo switching. **A,** Nogo task paradigm. In contrast to the Go task (upper panel), the rat must halt licking while within the judgement time window of 5 s to receive the reward (success). However, it is not rewarded if it licks the nozzle within the judgment time window (failure). **B,** Sample raster plots of the Go-Nogo switching test. The Go task conditioning (session O) was followed by sessions of Nogo tasks (I~V). The red squares indicate the timing of rewards, whereas the green squares the first licks without rewards. **C,** Cumulative probability plots of the reaction time for the data shown in B: sessions O~V. ^#^, P < 0.05 and ^##^, P < 0.005 (Kolmogorov-Smirnov test) compared to session O. **D,** Summary of agility through consecutive sessions (n = 7). **E**, Summary of failure rate (green marks) (n = 7). Although the lick within the judgement time window (5 s) after the light cue was judged as a success in session O (black symbol), it was judged as a failure in the following sessions (I~V). **F**, Summary of success rate (red marks) (n = 7). ^†^, P < 0.05, Wilcoxon signed rank test between failure and success rates. ^‡^, P < 0.05, ^‡‡^, P < 0.01, Kruskal-Wallice test by ranks.

Next, a Nogo task paradigm was so designed that the rat must halt licking for the judgement time window of 0–5 s to receive the reward (Fig 7A, success). However it was not rewarded if it licked the nozzle within the judgment time window (failure). When switched from the Go to Nogo task, the rat often licked with failure during early sessions (eg. session I of Fig 7B). However, it gradually learned the Nogo task and halted licking until receiving the reward (eg. session V of Fig 7B). For the Nogo task, the reaction time was measured only when the rat was not rewarded because it was prompted to lick upon rewarding (Fig 7C). As shown in Fig 7D, this behavioral change was quantitatively evaluated by the agility: it was relatively large at the beginning of Nogo task sessions (I and II) but significantly small at later sessions (III~V). Although the probability of the rat to lick during the judgement window (success rate) was over 80% during the Go task session, it was significantly reduced during session III~V of Nogo task (failure rate, Fig 7E). With the progress of the Nogo task sessions, once decreased during session I and II, the success rate was increased during later sessions (IV and V) with significant differences against the failure rate (Fig 7F).

### Spatiotemporally patterned cues

**Fig 8:**
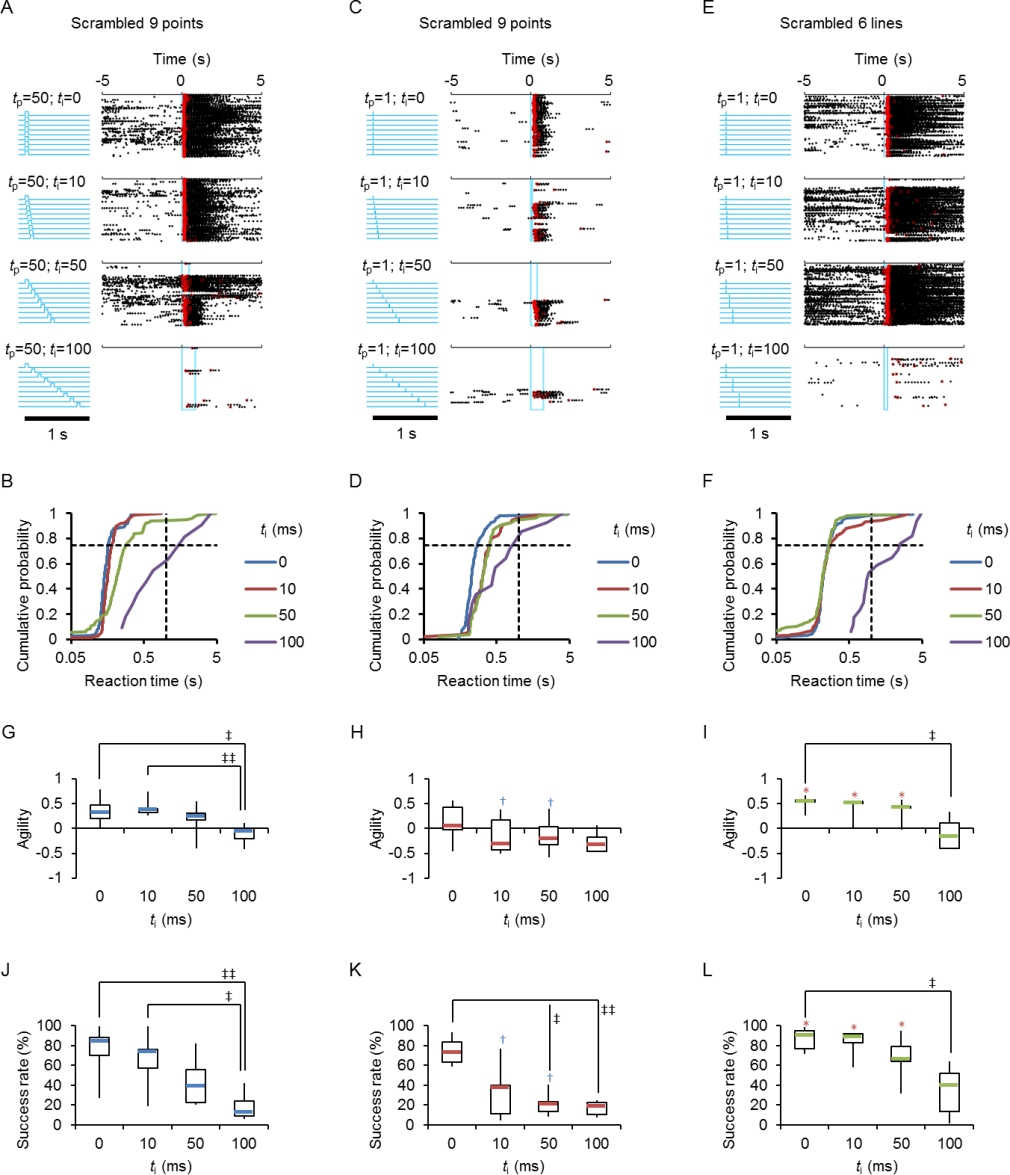
Spatiotemporally patterned cues. **A,** Each of 9 spots in the 3×3 matrix was irradiated simultaneously (*t*_i_ = 0) with a light pulse duration (*t*_p_) of 50 ms to establish the Go task conditioning (top panels). The behavioral responses were then tested by irradiating one-by-one (scrambled order) with an inter-pulse interval (*t*_i_) of 10, 50 and 100 ms (the second to fourth panels from the top). The blue vertical lines indicate the start and end of the cue irradiation. **B,** Cumulative probability plots of the reaction time for the data shown in A. **C,** The same rat was used for another test with *t*_p_ of 1 ms and variable *t*_i_ (0, 10, 50 and 100 ms from top to bottom). **D,** Similar to B, but for the data shown in C. **E,** Another rat was tested by the lined pattern, in which 3 spots aligned horizontally or vertically were irradiated simultaneously for 1 ms, and each of the 3 lined irradiation patterns was applied in a scrambled order with a *t*_i_ of 0, 10, 50 and 100 ms (from top to bottom). **F,** Similar to B, but for the data shown in E. **G-I,** Summary of the inter-pulse interval (*t*_i_) dependency of agility; the light pulse duration (*t*_p_) of 50 ms (G, blue, n = 7), 1 ms (H, red, n = 7) and lined pattern (I, green, n = 5). **J-L**, Similar to G-I, but a summary of the success rate. ^†^ (blue), P < 0.05, Wilcoxon signed rank test between scrambled (*t*_p_ = 50) and scrambled (*t*_p_ = 1). * (red), P < 0.05, Mann-Whitney *U*-test between scrambled (*t*_p_ = 1) and lined (*t*_p_ = 1). ^‡^, P < 0.05, ^‡‡^, P < 0.01, Kruskal-Wallice test by ranks.

To investigate the relationship between the spatiotemporal pattern of sensory inputs and the behavioral output, we designed a multi-optical fiber system that irradiates the whisker area at 9 spots in a 3×3 matrix, in each of which the timing of the irradiation is controlled independently. At first, the ChR2 rat was trained with Go tasks with synchronous irradiation of 9 spots with the light pulse duration (*t*_p_) of 50 ms to establish the conditioning (Fig 8A). Then the timing of the irradiation at the 9 positions was scrambled with an inter-pulse interval (*t*_i_) of 10, 50 and 100 ms. Although the rat licked the nozzle immediately and robustly with a *t*_i_ of 10 ms, it behaved rather awkwardly when *t*_i_ was increased to 100 ms. In this case, the rat stopped licking as a trait often observed when the cue was unpaired with the reward (e.g., Fig 2C, Fig 5A, Fig 6E, etc.). The change of the behavioral response was quantitatively expressed as a distributional shift of the reaction time (Fig 8B). Indeed, the agility was significantly decreased with a *t*_i_ of 100 ms (Fig 8G). Similarly, the success rate was significantly decreased when *t*_i_ was increased to 100 ms (Fig 8J).

Next, the behavioral outputs were evaluated for similar protocols with *t*_p_ of 1 ms (Fig 8C). Although the licking behavior was conditioned to the light cue, the response was awkward even with *t*_i_ of 10 ms (Fig 8D). The agility was significantly smaller than the corresponding responses to large *t*_p_ (50 ms) and the success rate was significantly reduced when *t*_i_ was increased to 10 ms (Fig 8H and 8K). In contrast, when the lined pattern, in which the 3 spots (*t*_p_ = 1 ms) aligned horizontally or vertically were irradiated simultaneously, was applied in a scrambled order with an *t*_i_ of 0, 10 or 50 ms, the first lick was synchronized to the light cue (Fig 8E and 8F). However, the licking behavior became awkward with a *t*_i_ of 100 ms. As summarized in Fig 8I and 8L, the agility and the success rate were significantly decreased when *t*_i_ was 100 ms.

### Robustness of conditioned behavior

**Fig 9:**
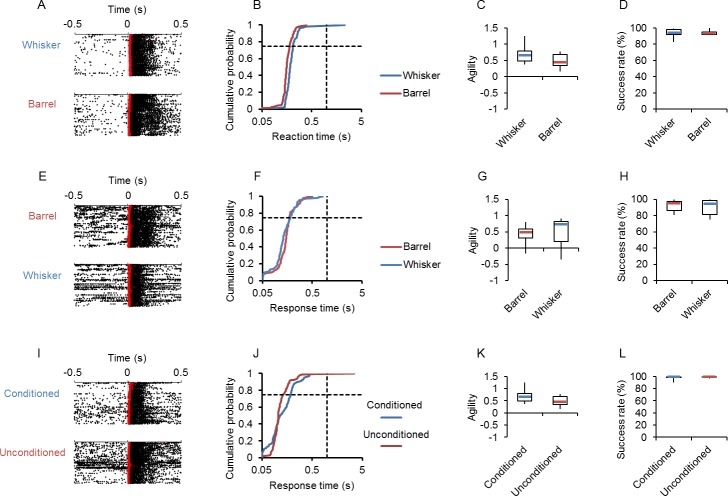
Robustness of conditioned behavior. **A,** Sample raster plots of cue-aligned licking events for the conditioned photostimulation of right whisker area (top) and for the unconditioned irradiation of the barrel region of the left cortex (bottom). **B,** Cumulative probability plots of the reaction time for the data shown in A; the whisker area photostimulation (blue) and the contralateral barrel irradiation (red). **C,** Summary of agility (n = 6). **D**, Summary of success rate (n = 6). **E,** Sample raster plots of cue-aligned licking events for the conditioned irradiation of the barrel region of the left cortex (top) and for the unconditioned photostimulation of the right whisker area (bottom). **F,** Cumulative probability plots of the reaction time for the data shown in E; the barrel irradiation (red) and the contralateral whisker area photostimulation (blue). **G,** Summary of agility (n = 6). **H**, Summary of success rate (n = 6). **I,** Sample raster plots of cue-aligned licking events for the conditioned photostimulation of the right whisker area (top) and for the unconditioned photostimulation of the left whisker area. **J,** Cumulative probability plots of the reaction time for the data shown in A; the conditioned whisker area photostimulation (blue) and the unconditioned contralateral photostimulation (red). **K,** Summary of agility (n = 6). **L**, Summary of success rate (n = 6).

The above observation suggests that the synchronous activation of the S1 region would be efficiently recognized as a conditioning cue (see Discussion). Since many cortical neurons, both excitatory and inhibitory, express ChR2 in W-TChR2V4 rat [17,19], the direct blue irradiation on the barrel region is expected to evoke the synchronous activation of neurons without topographical specification. Indeed, the direct irradiation on the contralateral barrel region was robustly accompanied by licking, which had been conditioned to photostimulation of the whisker area with a similar reaction time (Fig 9A and 9B). Although variable among experiments, no significant differences in the agility as well as the success rate were found between the conditioned whisker photostimulation and the unconditioned barrel irradiation (Fig 9C and 9D). Conversely, the ChR2 rat was conditioned to direct photostimulation of the barrel cortex with blue LED light as a cue to respond by licking within the judgement period of 5s (Fig 9E, top). Although the rat was naïve to the whisker area photostimulation, it robustly reacted to the irradiation on the contralateral whisker area with licking as early as the reaction to the conditioning cue (Fig 9E, bottom and 9F). Again, no significant differences in the agility as well as the success rate were found between the conditioned barrel irradiation and the unconditioned whisker photostimulation (Fig 9G and 9H).

We next examined whether the rat conditioned with the photostimulation of the whisker area of one side (usually, right) is responsive to photostimulation of the contralateral (left) whisker area (Fig 9I). Although unconditioned previously, the contralateral photostimulation was robustly accompanied with the licking behavior with a similar reaction time (Fig 9J). No significant differences were found in the agility and the success rate between whisker photostimulations of the conditioned side (right) and of the unconditioned side (left) (Fig 9K and 9L).

## Discussion

In the present study we irradiated blue light on the whisker area as a cue to condition the behavior of W-TChR2V4 rats that express ChR2 in the mechanoreceptive neurons in the TG. It is suggested that the cue is a kind of mechanoreception optogenetically evoked in the nerve endings based on the following four facts. First, the same training protocol never succeeded in inducing conditioning with the Go task in the wild-type rats. Secondly, the W-TChR2V4 rats learned to lick the water nozzle with high frequently after the blue light cue as the training sessions progressed even after the deprivation of bilateral vision. Third, the blue-light conditioned W-TChR2V4 rats did not respond with licking at all to the red light cue, which is not sensed by ChR2. Fourthly, the blue-light conditioned W-TChR2V4 rats no longer associated the blue light cue with its behavior when the whisker area was regionally shaded. Therefore, the possibility that other sensory stimuli such as leaked blue light into the eye or the pump sound were serving as cue signals could be excluded.

Although dependent on the irradiance, the activation of the ChR2 photocurrent was very fast and reached its peak within several ms [7,8]. When the membrane potential is depolarized, the latency to evoke an action potential is also determined by the membrane properties of the neuron. Indeed, a single action potential was evoked in 1–10 ms in the ChR2-expressing TG neurons from the W-TChR2V4 rat [11]. As a result, the optogenetic inputs on the whisker area arrive at the somatosensory cortex synchronously within 20 ms from their onset. This enabled us to evaluate the establishment of conditioning by measuring the time between the light cue onset and the first licking response thereafter (reaction time), the majority of the time serving for decision making [20]. The reaction time is expected to be decreased when the animal’s decision is rewarded and increased when not. Indeed, its distribution and the agility showed a closed relationship to the presence/absence of the rewards. However, the reaction time was also increased when the paradigm was changed from Go to Nogo and the rat successfully rewarded with halting lick for the designed period. It is possible that the sensory information was also stored in working memory, then used for decisions under the Nogo paradigm [21].

One of the advantages of optogenetics is its high spatiotemporal precision of neural activation. Indeed, the rat conditioned with the synchronous irradiation of 9 spots could not evoke timed licks when the same spots were irradiated asynchronously. The difference between synchronous and asynchronous irradiation was more obvious when the light pulse duration was reduced to 1 ms from 50 ms. However, it also depended on the number of spots irradiated simultaneously. Because of the activation kinetics of ChR2, each peripheral nerve should be more depolarized at the membrane by 50 ms light than by 1 ms [8]. As a result, the longer the light pulse, the greater the chance would be of a nerve generating an action potential. Similarly, more nerves were activated when 3 spots were irradiated synchronously at the same time in a lined pattern. It is suggested that a kind of neural circuit is present in the somatosensory cortex that detects the synchrony of a certain number of sensory inputs and the activation of this circuit is efficiently recognized as a conditioning cue. This is consistent with the fact that the establishment of learning was very fast (within 4 hours) in the present optogenetic system, whereas training for a few days was necessary to establish the whisker-dependent conditioning in the previous studies [6,12,22,23]. Probably, the activation of this circuit may not be dependent on the topographical features of inputs since the conditioned behavior cued by the whisker area photostimulation was also evoked by direct light stimulation of the barrel cortex and *vice versa*. However, the CNS circuitry activated by synchronous inputs should overlap with that activated by asynchronous inputs because the rat conditioned by asynchronous photostimulation of the whisker area also responded to the synchronous inputs with a high agility and success rate (S2 Fig). Although thalamic inputs from the ventro-postero-medial nucleus (VPM) topographically innervate the barrel columns predominantly in layer 4 of the primary somatosensory cortex (S1) [24–26], the inputs from the medial portion of the posterior complex (POm) project broadly across barrels in layers 1, 5A and the septal regions between barrels [25–28]. The coordinated inputs from both pathways should activate a certain number of cortical neurons to evoke the behavioral response [29], although even a train of stimulation given in a single neuron in the rat barrel cortex was detected as a cue for behavioral conditioning [30]. Irrespective to the actual neural pathways involved, it is plausible that the synchronous activation of a number of mechanoreceptive nerves is mimicking the sense of touch for a large object, one of the emergent alerts for a rat.

Another novel finding is that the licking behavior conditioned to the photostimulation on one side of the whisker area is robustly evoked by photostimulation on another side without previous conditioning. These two responses were almost symmetrical; there was no difference in the reaction time as well as the success rate between photostimulations of conditioned and unconditioned sides. Therefore, the neural circuits involved in this conditional behavior should be symmetrical between the left and right hemispheres. First, the sensory perception circuitry may be symmetrical; the signal into S1 of one side is transmitted to another side and *vice versa* to form a reverberating circuit between both sides [31]. Indeed, the layer 2/3 pyramidal neurons, which receive inputs from the layer 4 neurons, and the layer 5B pyramidal neurons, which receive thalamic VPM inputs, project cortico-cortically to the contralateral S1 [3,32–34]. Secondly, the decision may be made bilaterally to integrate the S1 activities at both sides. Finally, the motor outputs should be coordinated between both sides to evoke the licking behavior. Although the precise flow of signals underlying this symmetrical conditioning remains to be elucidated, the thalamo-cortical inputs and the layer-to-layer connections in S1 can be assumed not to be the primary sites of plasticity for the establishment of conditioning. Rather, the decision circuit that integrates the signal from both S1 and the top-down reward signal could be the site of plasticity, which was retained for days without entrainment.

In conclusion, we have developed a new model system of behavioral tests using optogenetics. Although artificial, the mechanoreceptive nerves at the whisker area were stimulated under spatiotemporal precision. Since ChR2 is exclusively expressed in the large mechanoreceptive neurons in DRG and TG [10,11], the photostimulation is presumed not to be accompanied by the sense of pain, which would produce unpredictable effects on the behavior. Our optogenetic system would enable one to evaluate precisely the psychophysical values, such as the reaction time and the success rate, to gain some insight into the brain mechanisms underlying conditioned behaviors.

## Supporting information

S1 Fig Statistical evaluation of the values. **A,** Distribution of “agility” in the cumulative probability plot. The gray line indicates the predicted normal distribution. The cue-responsiveness of rats was sensitively evaluated by the time when the cumulative probability reached to 0.75 (the third quartile of reaction time, *RT*_75_) because it was almost larger than 1 s in the control groups (Wt and Up), but became less than 1 s with the establishment of conditioning. Although this value was skewed in the distribution, its logarithmic reciprocal, -log(*RT*_75_), which we named “agility” followed a mostly normal distribution in the control groups (P = 0.1898, Shapiro-Wilk normality test). Note that 99% of the agility was negative throughout the sessions of all rats in the control groups. **B-E,** The Spearman's rank correlation between the agility and the licking frequency in the control wild-type rat (**B**, Wt), the control W-TChR2V4 (ChR2) rat unpaired with reward (**C**, Up), the ChR2 rat paired with reward (**D**, ChR2) and the visually deprived ChR2 rat (**D**, VD), respectively. The licking frequency was measured between -5 and 0 s of the cue. **F,** Distribution of success rate in cumulative probability plot. Its distribution deviated little from the normal distribution (gray line) although the difference was statistically significant (P = 0.000575, Shapiro-Wilk normality test). **G-J,** Similar to B-E, but the Spearman's rank correlation between the success rate and the licking frequency. It is suggested that both values, the agility and the success rate, would be independent of the preceding licking frequency.
(PDF)

S2 Fig Conditioning by the asynchronous inputs. **A,** Each of 9 spots in 3×3 matrix was irradiated one-by-one (scrambled order) with an inter-pulse interval (*t*_i_) of 100 ms with a light pulse duration (*t*_p_) of 50 ms to establish the Go task conditioning (raster plot); the blue vertical lines indicate the start and end of cue irradiation. **B,** The behavioral responses were then tested by irradiating simultaneously (*t*_i_ = 0). **C,** Cumulative probability plots of the reaction time for the data shown in A (red line, *t*_i_ = 100 ms) and B (blue line, *t*_i_ = 0 ms). **D,** Summary of the agility as the dependency to the inter-pulse interval (*t*_i_) (n = 5). **E,** Summary of the success rate as the dependency to the inter-pulse interval (*t*_i_) (n = 5).
(PDF)
