# Supplementary material for: Optogenetic conditioning of paradigm and pattern discrimination in the rat somatosensory system
Source: PLoS One. 2017 Dec 21;12(12):e0189439. doi: 10.1371/journal.pone.0189439 (PMC5739416; doi:10.1371/journal.pone.0189439)
Supplement: S1 Fig — A, Distribution of “agility” in the cumulative probability plot. The gray line indicates the predicted normal distribution. The cue-responsiveness of rats was sensitively evaluated by the time when the cumulative probability reached to 0.75 (the third quartile of reaction time, RT75) because it was almost larger than 1 s in the control groups (Wt and Up), but became less than 1 s with the establishment of conditioning. Although this value was skewed in the distribution, its logarithmic reciprocal, -log(RT75), which we named “agility” followed a mostly normal distribution in the control groups (P = 0.1898, Shapiro-Wilk normality test). Note that 99% of the agility was negative throughout the sessions of all rats in the control groups. B-E, The Spearman's rank correlation between the agility and the licking frequency in the control wild-type rat (B, Wt), the control W-TChR2V4 (ChR2) rat unpaired with reward (C, Up), the ChR2 rat paired with reward (D, ChR2) and the visually deprived ChR2 rat (D, VD), respectively. The licking frequency was measured between -5 and 0 s of the cue. F, Distribution of success rate in cumulative probability plot. Its distribution deviated little from the normal distribution (gray line) although the difference was statistically significant (P = 0.000575, Shapiro-Wilk normality test). G-J, Similar to B-E, but the Spearman's rank correlation between the success rate and the licking frequency. It is suggested that both values, the agility and the success rate, would be independent of the preceding licking frequency. (PDF) [file pone.0189439.s001.pdf]

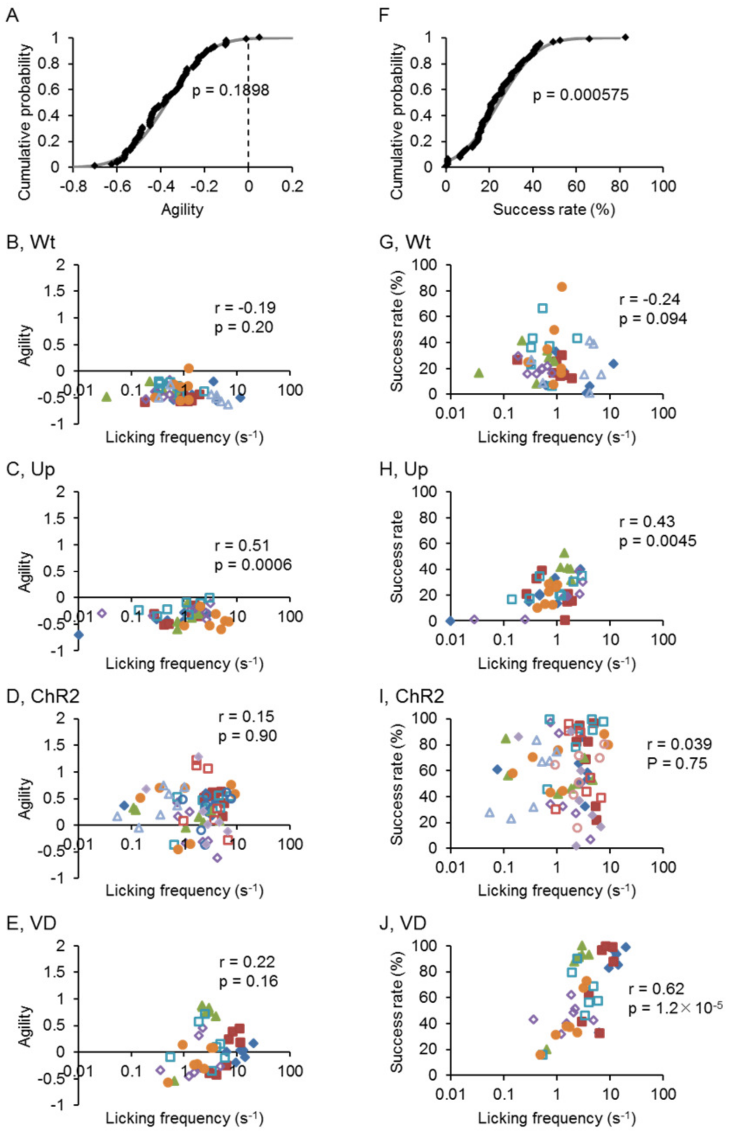

S1 Fig. Statistical evaluation of the values.

A, Distribution of "agility" in the cumulative probability plot. The gray line indicates the predicted normal distribution. The cue-responsiveness of rats was sensitively evaluated by the time when the cumulative probability reached to 0.75 (the third quartile of reaction time, RT_{75}) because it was almost larger than 1 s in the control groups (Wt and Up), but became less than 1 s with the establishment of conditioning. Although this value was skewed in the distribution, its logarithmic reciprocal, -log(RT_{75}), which we named "agility" followed a mostly normal distribution in the control groups (P = 0.1898, Shapiro-Wilk normality test). Note that 99% of the agility was negative throughout the sessions of all rats in the control groups. **B-E,** The Spearman's rank correlation between the agility and the licking frequency in the control wild-type rat (**B**, Wt), the control W-TChR2V4 (ChR2) rat unpaired with reward (**C**, Up), the ChR2 rat paired with reward (**D**, ChR2) and the visually deprived ChR2 rat (**D**, VD), respectively. The licking frequency was measured between -5 and 0 s of the cue. **F,** Distribution of success rate in cumulative probability plot. Its distribution deviated little from the normal distribution (gray line) although the difference was statistically significant (P = 0.000575, Shapiro-Wilk normality test). **G-J,** Similar to B-E, but the Spearman's rank correlation between the success rate and the licking frequency. It is suggested that both values, the agility and the success rate, would be independent of the preceding licking frequency.
