# Supplementary material for: Optogenetic conditioning of paradigm and pattern discrimination in the rat somatosensory system
Source: PLoS One. 2017 Dec 21;12(12):e0189439. doi: 10.1371/journal.pone.0189439 (PMC5739416; doi:10.1371/journal.pone.0189439)
Supplement: S2 Fig — A, Each of 9 spots in 3×3 matrix was irradiated one-by-one (scrambled order) with an inter-pulse interval (ti) of 100 ms with a light pulse duration (tp) of 50 ms to establish the Go task conditioning (raster plot); the blue vertical lines indicate the start and end of cue irradiation. B, The behavioral responses were then tested by irradiating simultaneously (ti = 0). C, Cumulative probability plots of the reaction time for the data shown in A (red line, ti = 100 ms) and B (blue line, ti = 0 ms). D, Summary of the agility as the dependency to the inter-pulse interval (ti) (n = 5). E, Summary of the success rate as the dependency to the inter-pulse interval (ti) (n = 5). (PDF) [file pone.0189439.s002.pdf]

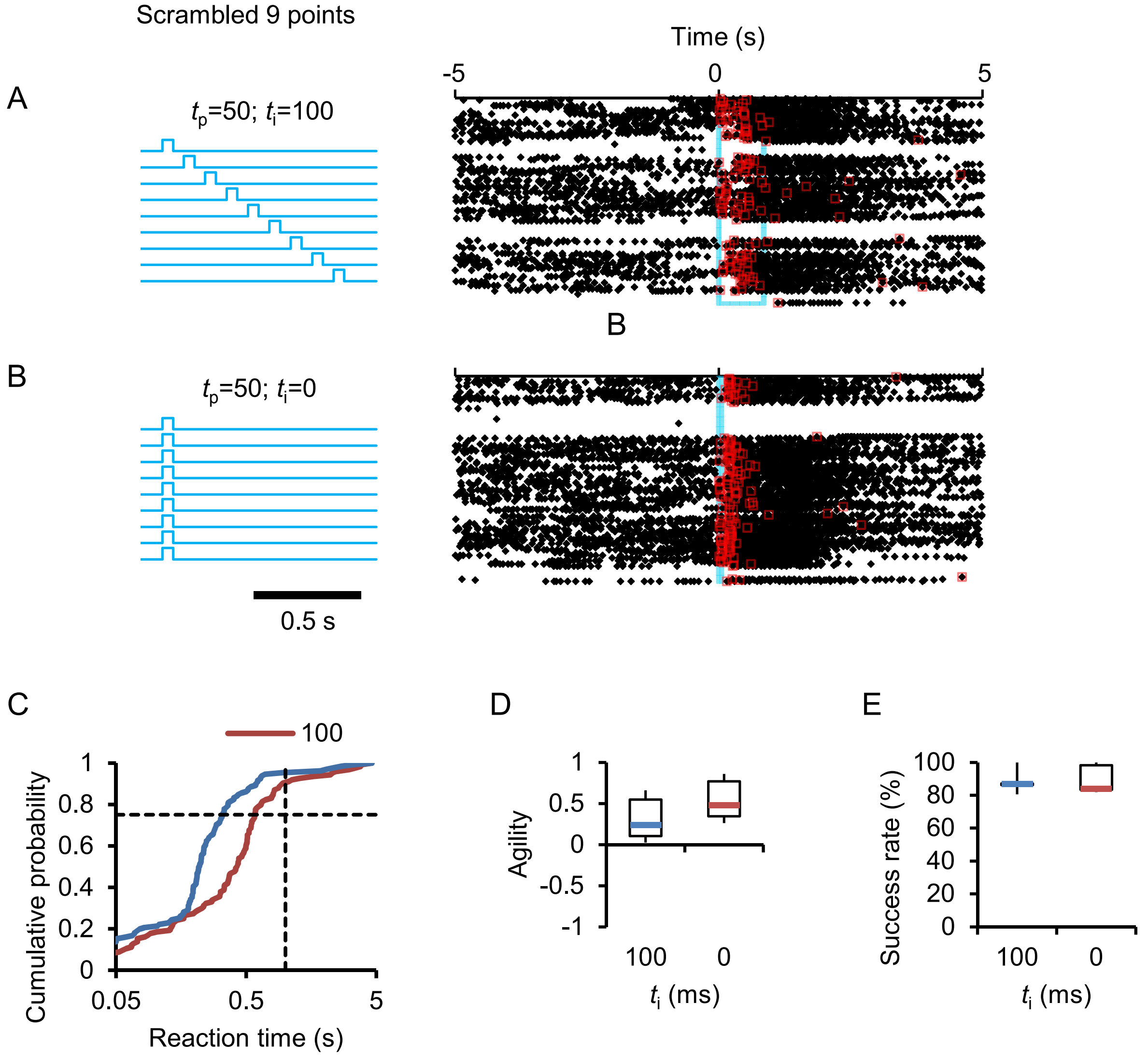

S2 Fig. Conditioning by the asynchronous inputs. A, Each of 9 spots in 3 × 3 matrix was irradiated one-by-one (scrambled order) with an inter-pulse interval (t_i) of 100 ms with the light pulse duration (t_p) of 50 ms to establish the Go task conditioning (raster plot); the blue vertical lines indicate the start and end of cue irradiation. **B,** The behavioral responses were then tested by irradiating simultaneously (t_i = 0). **C,** Cumulative probability plots of the reaction time for the data shown in A (red line, t_i = 100 ms) and B (blue line, t_i = 0 ms). **D,** Summary of agility as the dependency to the inter-pulse interval (t_i) (n = 5). **E,** Summary of success rate as the dependency to the inter-pulse interval (t_i) (n = 5).
